# The novel organoselenium compound 4aa ameliorates osteoporosis by modulating gut microbiota composition and fecal metabolite profiles

**DOI:** 10.3389/fendo.2025.1623933

**Published:** 2025-08-13

**Authors:** Chaoming Hu, Yichi Zhang, Yao Wu, Junhao Tu, Mengjia Yi, Yixin Mao, Yang Chen, Xiaoyu Sun, Zengqiang Song, Shengbin Huang, Shufan Zhao, Bin Li

**Affiliations:** ^1^ Institute of Stomatology, School and Hospital of Stomatology, Wenzhou Medical University, Wenzhou, China; ^2^ School of Pharmaceutical Sciences, Wenzhou Medical University, Wenzhou, China; ^3^ Department of Prosthodontics, School and Hospital of Stomatology, Wenzhou Medical University, Wenzhou, China; ^4^ Department of Periodontics, School and Hospital of Stomatology, Wenzhou Medical University, Wenzhou, China; ^5^ Department of Oral Maxillofacial Surgery, School and Hospital of Stomatology, Wenzhou Medical University, Wenzhou, China

**Keywords:** osteoporosis, β-trifluoroethoxy dimethyl selenide, gut microbiota, gut metabolites, α-KIV

## Abstract

**Background:**

The gut microbiota plays a key role in regulating bone homeostasis. Our previous work demonstrated that the novel organic selenium compound β-trifluoroethoxy dimethyl selenide (4aa alleviates osteoporosis; however, its mechanism remains unclear.

**Method:**

The cytotoxicity of 4aa in osteoblast (MC3T3-E1) and osteoclast precursor (RAW264.7) cells was evaluated using CCK-8 assays. Ovariectomized (OVX) and sham-operated mice were treated with various concentrations of 4aa for 8 weeks, including a subgroup pretreated with antibiotics (ABX) to deplete the gut microbiota. Femoral bone structure was assessed by micro-computed tomography (micro-CT), osteoclast numbers were quantified, gut microbial composition was analyzed via 16S rRNA sequencing, and fecal metabolites were profiled using LC-MS/MS.

**Results:**

4aa concentrations below 20 μM were non-cytotoxic to MC3T3-E1 and RAW264.7 cells. *In vivo*, 4aa significantly improved femoral bone mass and trabecular microarchitecture in OVX mice. Gut microbiota analysis revealed increased relative abundances of *Dubosiella*, *Akkermansia*, and *Bacillus* spp following 4aa administration. Metabolomic profiling identified marked alterations in citronellal, tyrosol, kaempferol, leukotriene D4, clomipramine, and phenol sulfate level. Moreover, 4aa elevated butyric acid levels and reduced the accumulation of α-ketoisovaleric acid (α-KIV), contributing to the inhibition of osteoclast differentiation.

**Conclusion:**

4aa prevents estrogen deficiency-induced bone loss by modulating gut microbial composition and function. These findings support the therapeutic of 4aa as a microbiota-targeted therapeutic strategy for osteoporosis management.

## Background

Osteoporosis is a prevalent metabolic bone disorder characterized by reduced bone mineral density (BMD) and impaired bone quality, leading to increased fracture risk and considerable morbidity and mortality (1). Current therapeutic strategies, including bisphosphonates, estrogens and their receptor modulators, and calcitonin, are effective but have notable limitations (2–4). These drawbacks underscore the urgent need for safer, more effective anti-osteoporotic agents suitable for long-term use without significant adverse effects.

Emerging evidence highlights the critical role of the gut microbiome and its metabolites in bone metabolism, particularly in the context of osteoporosis (5, 6). Clinical studies suggest that the gut microbiota may reduce osteoporosis risk by modulating calcium absorption, immune function, and central nervous system activity (7–9). For example, segmented filamentous bacteria (SFB) can influence bone density by altering the proportion of immune cells, such as Th17 cells (10), while lactobacilli can enhance calcium bioavailability either by directly facilitating calcium absorption or by modifying dietary substrates (11). Additionally, gut-derived metabolites-such as short-chain fatty acids (SCFAs) and trimethylamine-N-oxide (TMAO)-may exert protective effects against osteoporosis via direct or indirect pathways (12–14). Thus, modulating the gut microbiota, either through pharmacologic alteration of microbial composition or metabolic regulation, presents a promising therapeutic avenue for osteoporosis prevention and treatment.

Due to its high hydrophobicity, the trifluoroethoxy group has been widely employed in antibacterial drug development to enhance molecular interactions with lipid membranes, thereby influencing drug distribution and permeability in biological systems (15). Selenium, a key antioxidant, plays an essential role in mitigating inflammation and maintaining bone health (16, 17), and selenium deficiency has been linked to Keshan and Kaschin-Beck diseases (18). Moreover, selenium supplementation has been shown to increase microbial diversity suggesting that selenium may simultaneously modulate gut microbiota and support bone health (19).

Building on these insights, our group synthesized a novel organic selenium compound-β-trifluoroethoxy dimethyl selenide (4aa)-which has been shown to prevent ovariectomy (OVX)-induced osteoporosis by inhibiting osteoclast differentiation and promoting osteoblast differentiation (20). However, the *in vivo* mechanism underlying the osteoprotective effects of 4aa remain unclear. Given selenium’s influence on gut microbes and non-antibiotic drugs (21). we hypothesized that 4aa may alter the intestinal microbiota and associated metabolites, thereby contributing to osteoporosis prevention *in vivo*.

This study aimed to investigate whether 4aa modulates gut microbiota and metabolic profiles in an OVX mouse model and to elucidate its potential mechanisms in preventing osteoporosis.

## Methods

### Cell culture

Osteoclast precursor cells (RAW264.7; ATCC, Manassas, VA, USA) were maintained in DMEM (SH30022.01; Thermo Fisher Scientific, Waltham, MA, USA) supplemented with 10% fetal bovine serum (FBS; Thermo Fisher Scientific, Waltham, MA, USA; 16,140,071) and 1% penicillin-streptomycin (Thermo Fisher Scientific, Waltham, MA, USA). In a similar manner, osteoblast precursor cells (MC3T3-E1; ATCC, Manassas, VA, USA) were maintained in α-minimum essential medium (Thermo Fisher Scientific, Gaithersburg, MD, USA) supplemented with 10% FBS and 1% penicillin-streptomycin. The cultures were incubated at 37°C in a humidified 5% CO_2_ atmosphere.

### BCAT1, BCAT2, and BCKDC Knockdown in RAW264.7 Cells

Stable knockdown of the murine Bcat1 (branched-chain amino acid transaminase 1), Bcat2 (branched-chain amino acid transaminase 2), and Bckde1a (branched-chain α-keto acid dehydrogenase E1 alpha subunit) genes in RAW264.7 macrophage cells was achieved using lentiviral vectors encoding gene-specific short hairpin RNA (shRNA). The shRNA target sequences were as follows: Bcat1: GGAACAGAGTGAAGGAGATGT; Bcat2: CCAAAGAACCACAGAAGAA; Bckde1a: GGGTACGGCATCATGTCAATC. Cells were infected with lentiviruses carrying the respective shRNA constructs and selected with 4 μg/mL puromycin for 7 days. Knockdown efficiency was confirmed at the transcript level by quantitative real-time PCR (qRT-PCR) and at the protein level by Western blot analysis using antibodies against BCAT1, BCAT2, and BCKDE1a. The primer sequences used for qRT-PCR are listed in **Table 1 of Supplementary Material 1**, and the antibody information is provided in **Table 2 of Supplementary Material 1**.

### Cell viability assay

Using a CCK-8 kit, the cytotoxicity of 4aa was measured (C6005; NCM Biotech, China). MC3T3-E1 or RAW264.7 cells were seeded in 96-well plates at a density of 10,000 cells per well and exposed to different concentrations of 4aa (0, 1, 5, 10, 20, 40, 80, and 160 μM) for 48 hours. Following this, 10 μL of CCK-8 solution was introduced to each well and left to incubate at 37°C for 30 minutes. Absorbance was then recorded using a microplate reader. (BioTek, Winooski, VT, USA).

### Osteoclast differentiation

RAW264.7 cells were induced to differentiate into osteoclasts using recombinant RANKL (462-TEC-01, R&D Systems, USA). Cells were seeded in 96-well plates at a density of 3,000 cells per well, as determined by manual counting. After 12 hours of incubation, the medium was replaced with differentiation medium containing 50 ng/ml RANKL. For nutrient-restriction experiments, amino acid–free DMEM (D9800-27, US Biological, USA) was used as the basal medium, with amino acid concentrations adjusted to match those of standard high-glucose DMEM (11965092, Thermo Fisher Scientific, USA). For drug treatment experiments, pharmacological compounds (**Table 3 in Supplementary Material 1**) were added at the time of medium replacement (12 hours after seeding) along with the differentiation medium, according to specific experimental requirements. Culture medium was refreshed every 48 hours to maintain drug efficacy. Detailed drug treatment protocols, including compound names and concentrations, are provided in the corresponding figure legends.

### TRAP staining

Tartrate-resistant acid phosphatase (TRAP) staining was performed to evaluate osteoclast differentiation. After the indicated treatment period, RAW264.7 cells were fixed with 4% paraformaldehyde for 10 minutes at room temperature and then rinsed with phosphate-buffered saline (PBS). TRAP staining was conducted using a commercially available Acid Phosphatase, Leukocyte (TRAP) Kit (387A-1KT, Sigma-Aldrich, USA), following the manufacturer’s instructions. Cells were incubated with the staining solution at 37°C for 4 hours in the dark. TRAP-positive multinucleated cells were quantified.

### Animals

Six-week-old female C57BL/6J mice were housed at the Animal Experimentation Center of Wenzhou University of Medical Sciences. Mice were given sterile food and autoclaved water freely available under a 12-hour light/dark cycle. To investigate the role of 4aa in preventing osteoporosis, 24 female mice underwent ovariectomy and randomly assigned to four groups: ovariectomy (OVX) group (N=6), and OVX + 4aa treatment groups receiving 5 mg/kg, 10 mg/kg, or 20 mg/kg of 4aa (N=6 per dose group). After confirming that 10 mg/kg of 4aa effectively alleviated osteoporosis symptoms in OVX mice, this dosage was selected for subsequent experiments. For sham-operated control, mice underwent the same surgical procedure except that neither the fallopian tubes were ligated nor the ovaries removed. The sham+4aa and OVX+4aa groups received intraperitoneal injections of 4aa (10 mg/kg) every other day for 8 weeks, while the other groups received normal saline injections. To further assess whether 4aa prevents osteoporosis via modulation of gut microbiota, eighteen more female C57BL/6J mice, aged six weeks, were randomly assigned to three groups: OVX group (N=6), OVX+ antibiotic solution treatment (OVX+ABX+4aa) group, and OVX+4aa treatment group (OVX+4aa). Mice in the OVX+ABX+4aa group received a drinking water solution containing antibiotics (ampicillin 0.1 mg/mL, streptomycin 0.5 mg/mL, and mucomycin 0.1 mg/mL) (22). The antibiotic solution was refreshed three times per week, alongside 4aa compound injections. The remaining groups were provided with autoclaved water. After the experimental period, femoral specimens were fixed in 4% paraformaldehyde for 48 hours. Microcomputed tomography (Micro-CT) was performed for analysis, and the results were evaluated.

### Micro-CT detection and analysis

Micro-CT analysis was performed on femoral growth plate specimens using the SkyScan1276 system (Bruker Corporation, Billerica, MA, USA) to evaluate differences in cancellous bone volume and structure among the four groups. The following parameters were quantified using the built-in software suite (NRecon, DataViewer, CTAn Version: 1.20.3.0): trabecular bone volume fraction (BV/TV), trabecular connectivity density (Conn. Dn), trabecular number (Tb. N), trabecular separation (Tb. Sp), and trabecular thickness (Tb. Th).

### Hematoxylin eosin stain

Bone samples from six mice were decalcified using 10% ethylenediaminetetraacetic acid (EDTA, pH 7.4; Solarbio, Beijing, China). Following decalcification, the samples underwent dehydration through a gradient ethanol series, followed by permeabilization and paraffin embedding. The specimens were then sectioned and stained with hematoxylin-eosin (HE; ZSGB-BIO, Beijing, China) to evaluate the condition of trabecular bone.

### 16S rRNA microbial community analysis

Total DNA was extracted from microbial communities in colon contents following the protocol of the E.Z.N.A.^®^ Soil DNA Kit (Omega Bio-tek, Norcross, GA, USA). The 16S rRNA gene’s V3-V4 region was amplified with primers 338F (5’-ACTCCTACGGGGAGGCAGCAG-3’) and 806R (5’-GGACTACHVGGGTWTCTAAT-3’). PCR (polymerase chain reaction) product sequencing was performed using the Illumina MiSeq PE300 platform. Representative operational taxonomic unit (OTU) sequences were annotated using the Silva 16S rRNA database (v138) with the RDP classifier, applying a confidence threshold of 0.7.

### Metabolite profile analysis

UHPLC-Q Exactive HF-X system (Thermo Fisher Scientific, Waltham, MA, USA) was used to conduct metabolite analysis. The raw data were preprocessed with Progenesis QI software (Waters Corporation, Milford, USA), and the ropls package (Version 1.6.2; Bioconductor, Seattle, WA, USA) in R was used for further data analysis. Time-series analysis of metabolomics data was conducted by averaging the six biological replicates within each group. The resulting metabolite profiles were subsequently analyzed and visualized using https://www.bioinformatics.com.cn, an online platform for omics data analysis and visualization (23).

### Statistical analyses

Each experiment was performed three times, and the outcomes are presented as the average ± standard deviation. One-way analysis of variance (13) with Fisher’s *post hoc* test was used for statistical analysis in GraphPad Prism software (GraphPad Software, San Diego, CA, USA). Statistical significance was defined as p<0.05, with levels of significance indicated as p<0.05, *p<0.01, and **p<0.001.

## Results

### Cytotoxicity of 4aa

**Figure 1:**
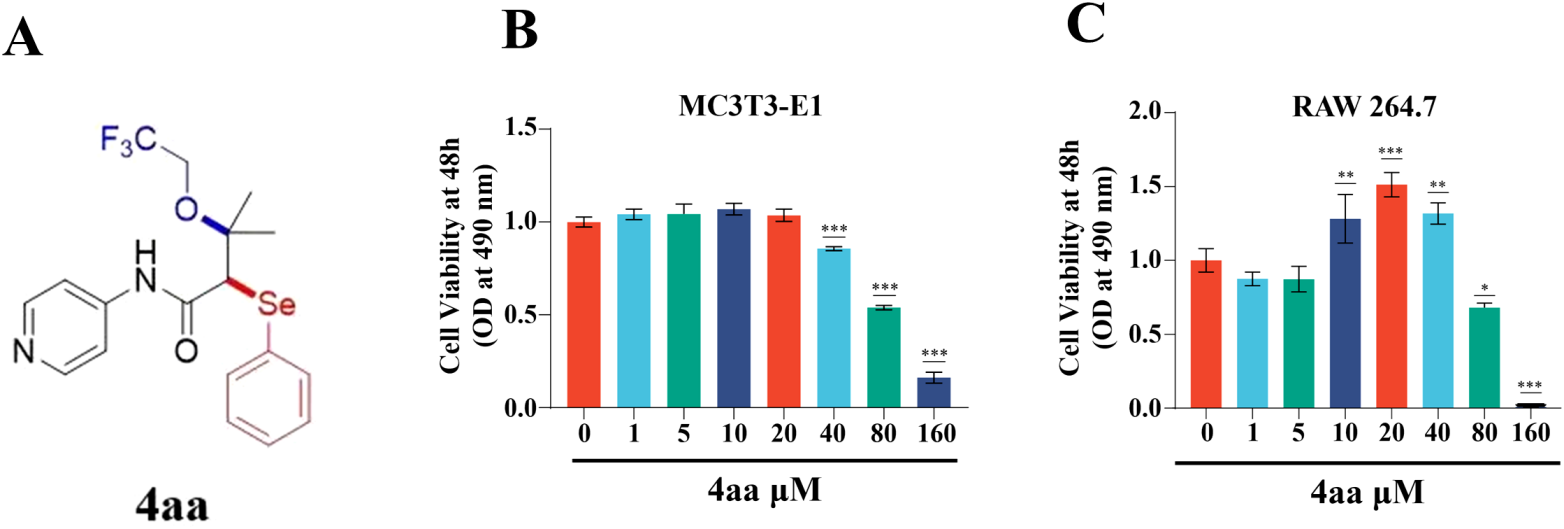
Assessment of 4aa Toxicity. **(A)** Structure of 4aa; **(B)** Cytotoxicity of 4aa in RAW264.7 cells; **(C)** Cytotoxicity of 4aa in MC3T3-E1 cells. *p < 0.05, **p < 0.01, and ***p < 0.001 vs control group.

Bone remodeling is a coordinated process involving bone formation by osteoblasts and bone resorption by osteoclasts (24). To evaluate the cytotoxic effects of 4aa on osteoblast precursor cells (MC3T3-E1) and osteoclast precursor cells (RAW264.7), cell viability was assessed using the CCK-8 assay (**Figures 1A-C**). Treatment with 4aa at concentrations ranging from 10 to 40 μM significantly promoted the proliferation of RAW264.7 cells (p<0.001); however, no proliferative effect was observed in MC3T3-E1 cells at the same concentrations (p<0.001). In contrast, concentrations above 80 µM exhibited cytotoxicity on both cell lines (p<0.001).

### 4aa inhibits bone loss in OVX mice model

**Figure 2:**
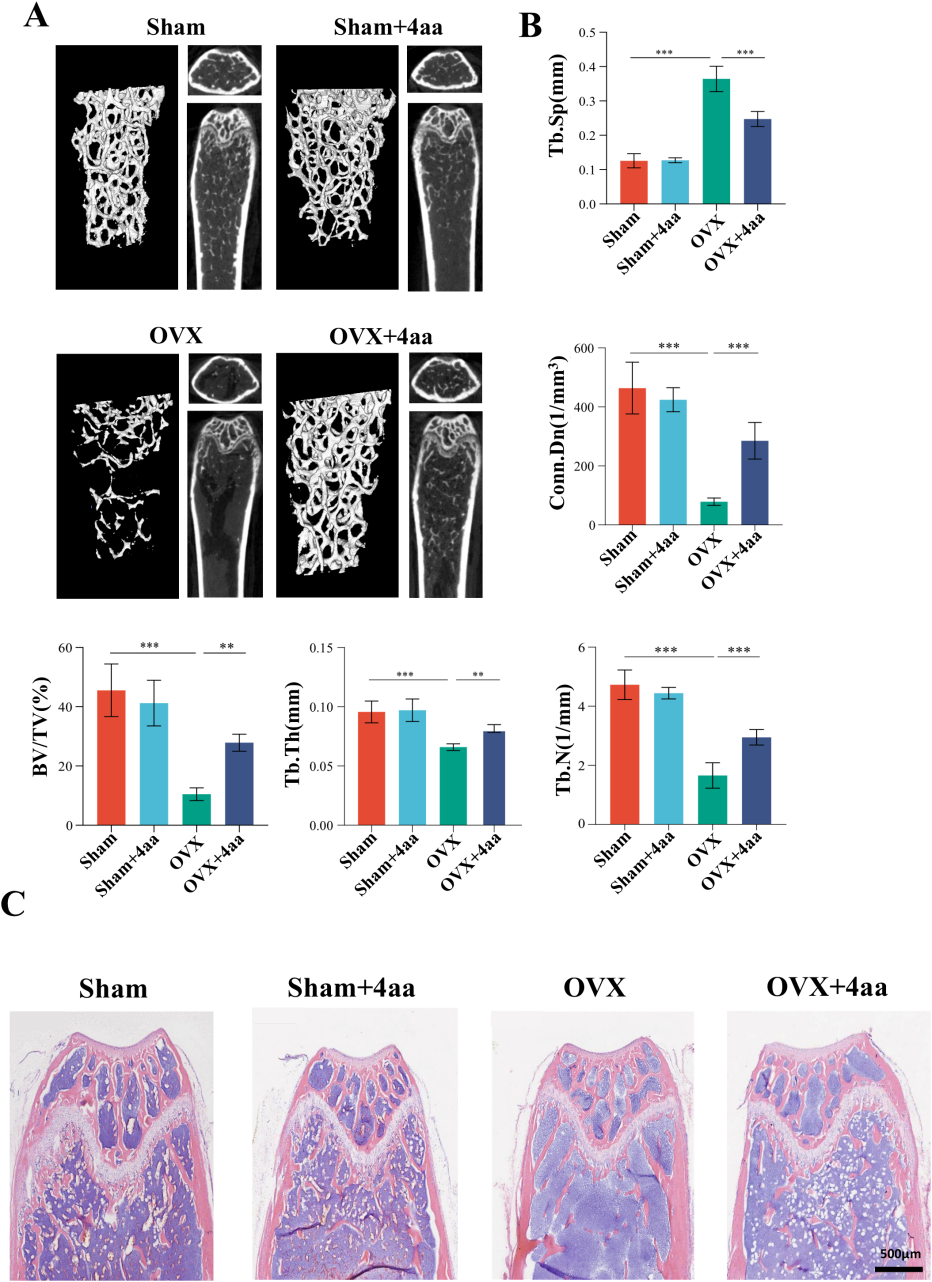
4aa treatment prevents bone loss and trabecular number reduction in OVX mice. **(A)** Representative 3D micro-CT images of femurs from sham, sham+4aa, OVX, OVX+4aa (10 mg/kg; N=6); **(B)** Quantification of trabecular bone volume fraction (BV/TV), trabecular junction density (Conn. Dn), trabecular number (Tb. N), trabecular separation (Tb.Sp), and trabecular thickness (Tb. Th) across groups (N=6); **(C)** Representative hematoxylin and eosin (H&E) staining of femoral sections (N=6). **p < 0.01, and ***p < 0.001.

Among all treatment groups, the 10 mg/kg and 20 mg/kg 4aa groups demonstrated the thickest and densest trabecular structures (**Supplementary Figure S1A, B**). As no significant difference in bone microstructure was observed between these two doses, 10 mg/kg was selected for subsequent experiments (**Figure 2A**). In the 4aa+OVX group, key structural parameters-including trabecular thickness (Tb.Th), trabecular number (Tb.N), connectivity density (Conn.Dn), and bone volume fraction (BV/TV)-were significantly higher than those in the OVX group (**Figure 2B**). Moreover, trabecular separation (Tb.Sp) was reduced following 4aa treatment (**Figure 2B**). These findings suggest that 4aa effectively mitigates bone loss in OVX mice. Additionally, histological analysis of the femur showed a marked increase in trabecular number following 4aa treatment, consistent with the micro-CT results (**Figure 2C**), confirming the osteoprotective effect of 4aa in OVX-induced bone loss.

### 4aa alters gut microbiota diversity and community structure in OVX mice

**Figure 3:**
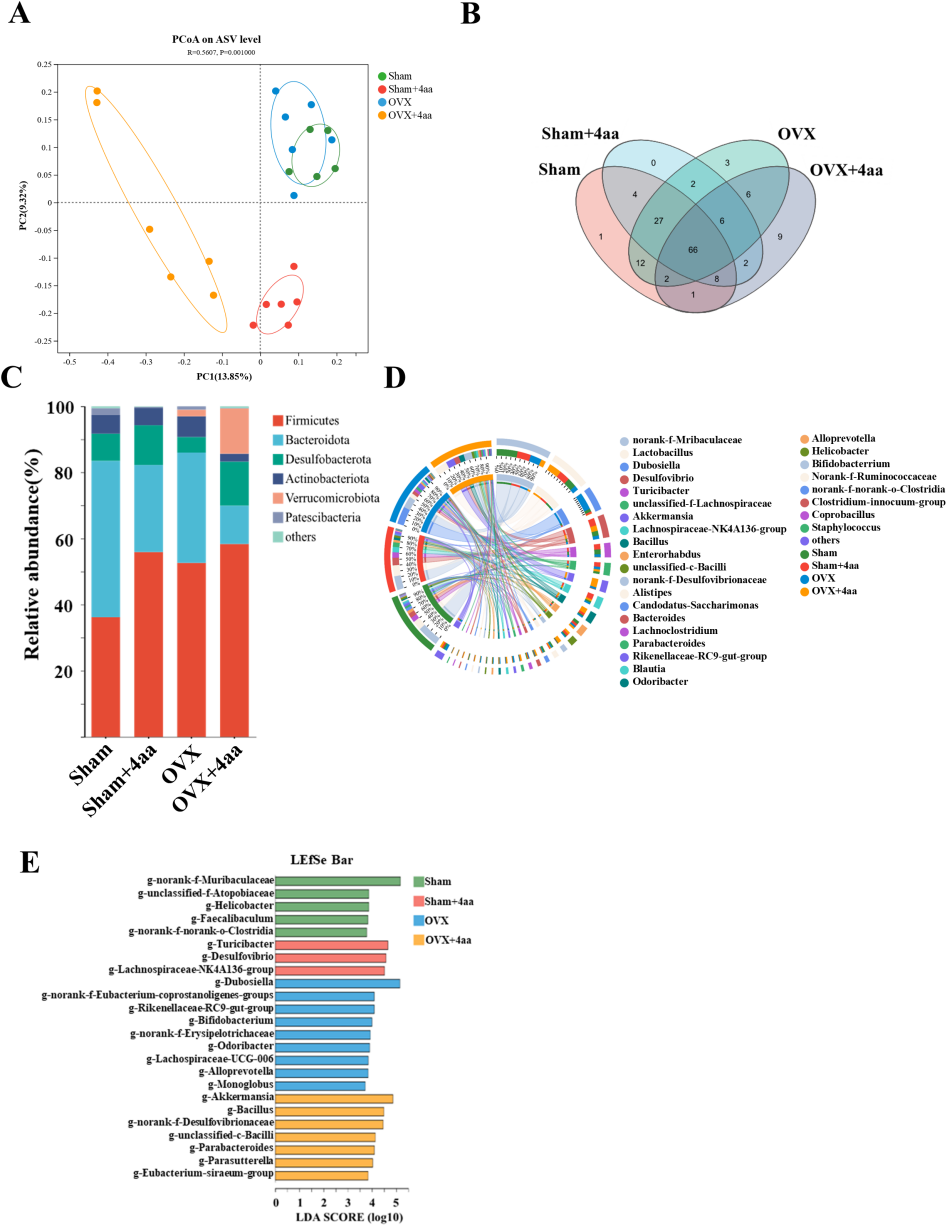
Alterations in gut microbial composition following 4aa treatment. **(A)** PCoA plot of microbiota composition (PC1 = 13.85%, PC2 = 9.32%). Green: Sham; Red: Sham+4aa; Blue: OVX group; Yellow: OVX+4aa group (N = 6); **(B)** Venn diagram of shared ASVs across groups; **(C)** Phylum-level microbial differences across groups (N=6); **(D)** Circos plot showing genus-level distribution across groups (N = 6); **(E)** Linear discriminant analysis (LDA) effect size (LEfSe) (LDA score ≥ 3.5). Color coding as above.

Given the known antimicrobial and anti-inflammatory properties of 4aa, we performed an amplicon sequence variant (ASV) analysis. Across all groups, a total of 3735 ASVs were identified, encompassing 11 phyla, 16 orders, 41 classes, 68 families, 139 genera, and 235 species. Principal coordinate analysis (PCoA) revealed distinct clustering patterns among groups, with statistical significance confirmed by PERMANOVA (R² = 0.5607, p = 0.001) (**Figure 3A**), indicating that 4aa injection significantly altered the gut microbiota.

The 16S rDNA sequencing revealed three unique ASVs in the OVX group, whereas nine unique ASVs emerged in the OVX+4aa group, suggesting substantial microbiota changes following 4aa treatment (**Figure 3B**). At the phylum level, an increase in *Desulfobacterota* was observed in both the sham and OVX groups after 4aa injection. Conversely, the abundance of *Bacteroidota* decreased following treatment in both groups (**Figure 3C**). Additionally, distinct microbiota profiles were evident in the OVX and OVX+4aa groups compared with the sham group (**Supplementary Figures S2A-E**). Notably, the abundance of *Verrucomicrobiota* significantly increased in the OVX+4aa group but was not detected in the sham group (**Supplementary Figure S2E**).

The genus *Dubosiella*, which was elevated in the OVX group, was significantly reduced after 4aa treatment (p<0.001), suggesting a potential deleterious association with OVX-induced dysbiosis (**Figure 3D**, **Supplementary Figure S2F**). In contrast, the abundances of *Akkermansia* (p<0.001) and *Ballus* (p<0.01) increased significantly after treatment, suggesting a possible beneficial role of these genera (**Supplementary Figures S2H, I**). Finally, linear discriminant analysis (LDA) with effect size estimation identified differentially abundant taxa with LDA scores > 3.5 across treatment groups (**Figure 3E**).

### Metabolomics analysis of fecal samples after 4aa treatment

**Figure 4:**
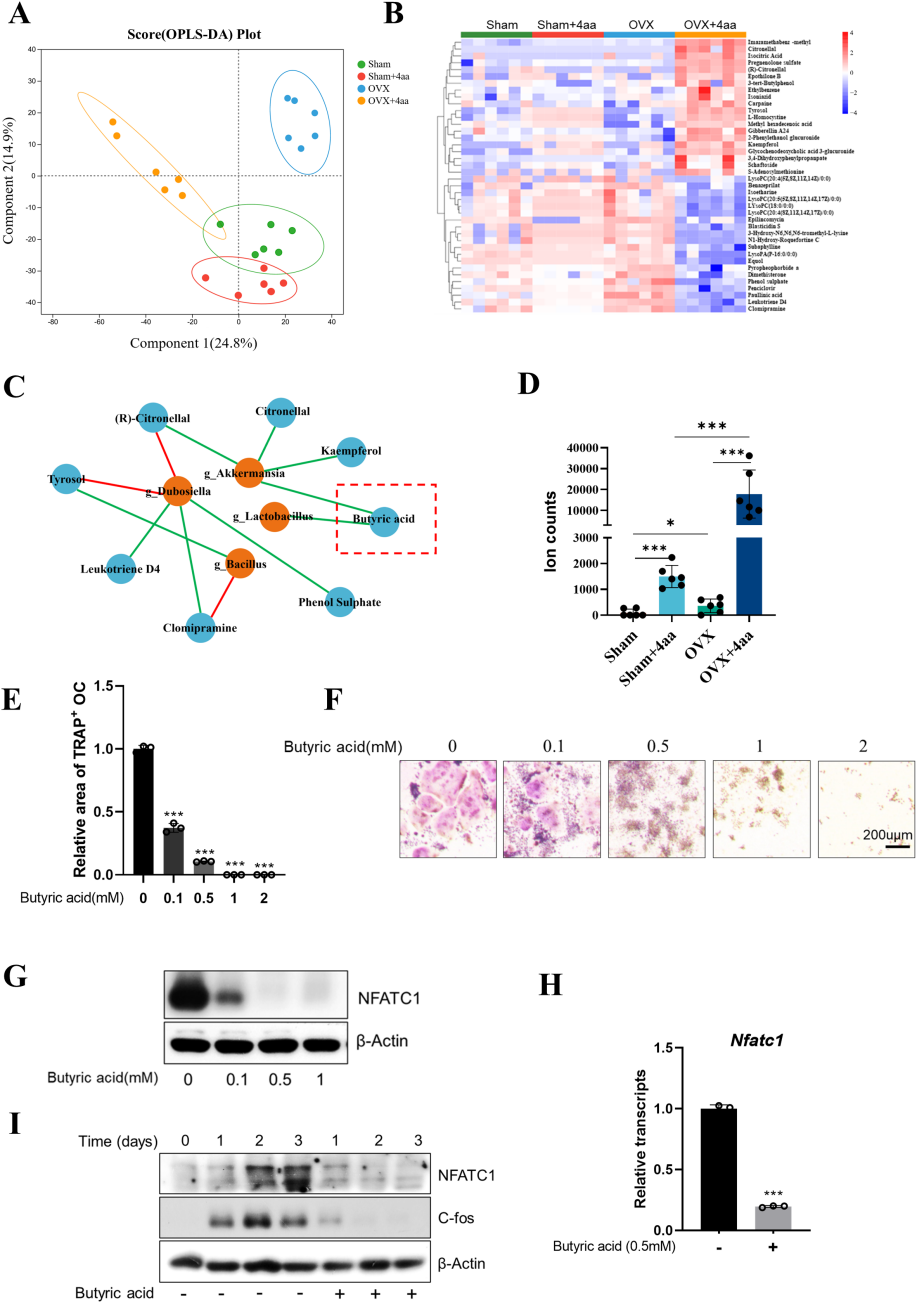
4aa treatment alters gut metabolites and suppresses osteoclastogenesis via butyric acid. **(A)** OPLS-DA plot of fecal metabolites; **(B)** Heatmap of differential fecal metabolites; **(C)** Correlation between bacteria taxa and fecal metabolites; **(D)** Butyric acid abundance in response to 4aa; **(E)** Quantification of TRAP staining in RAW264.7 cells treated with butyric acid; **(F)** Representative TRAP-stained cells; **(G)** NFATc1 protein expression following 3-day treatment with butyric acid; **(H)** NFATc1 mRNA levels after 3-day 0.5 mM butyric acid treatment; **(I)** Time-course of NFATc1 and c-Fos protein levels after 0.5 mM butyric acid treatment. *p < 0.05 and ***p < 0.001.

Changes in the composition and abundance of intestinal microbiota are often associated with alterations in gut-derived metabolites. Therefore, we performed a metabolomics analysis of intestinal contents across all groups. Orthogonal partial least squares discriminant analysis (OPLS-DA) revealed differences in colonic metabolite profiles between the OVX and OVX+4aa groups (**Figure 4A**). We identified the top 20 upregulated and downregulated metabolites (**Figure 4B**, **Supplementary Figures S3A, B**), including Citronellal, Kaempferol, Pregnenolone sulfate, Tyrosol, L-Homocystine, Gibberellin A24, Glycochenodeoxycholic acid 3-glucuronide, 2-Phenylethanol glucuronide, Isocitric Acid, and Methyl hexadecanoic acid, which were significantly elevated in the OVX+4aa group. Conversely, 10 metabolites-Epilincomycin, Leukotriene D4, Dimethisterone, Equol, Subaphylline, Phenol sulphate, Paullinic acid, Pyropheophorbide a, LysoPA (P-16:0/0:0), and Clomipramine-were significantly reduced following 4aa treatment.

### Correlation between gut microbiota and metabolome in OVX and OVX+4aa groups

As 4aa treatment led to significant alterations in both the gut microbiota and fecal metabolite composition (**Figures 4A, B**), we examined the relationship between bacterial genera and metabolite abundance. A heatmap was generated to visualize the associations between bacterial genera and key metabolites (**Supplementary Figure S4A**).

Specifically (R)-Citronellal (r=0.700), Kaempferol (r=0.760), Citronellal (r=0.704) and Butyric acid (r=0.710) were positively correlated with *Akkermansia* based on integrated fecal metabolomics and 16S rRNA gene sequencing. In contrast, Tyrosol (r=-0.868) and (R)-Citronellal (r=-0.833) were negatively correlated with *Dubosiella*. Additionally, Clomipramine (r=0.903), Leukotriene D4 (r=0.855), and Phenol sulfate (r=0.704) were positively correlated with *Dubosiella*, while Clomipramine (r=-0.759) and Tyrosol (r=0.700) were negatively and positively correlated, respectively, with *Bacillus* spp (**Figure 4C**).

We also observed direct associations between butyric acid and the genera *Akkermansia* and *Lactobacillus*. As a characterized short-chain fatty acid (SCFA), butyric acid has been reported to suppress osteoclast differentiation (25, 26). In our study, 4aa treatment significantly increased butyric acid levels in both sham and OVX groups (p< 0.001; **Figure 4D**). Furthermore, induction of osteoclast differentiation in the presence of various concentrations of butyric acid demonstrated a marked inhibitory effect, even at 0.1 mM (**Figures 4E, F**).

Previous studies have identified butyric acid as a histone deacetylase (HDAC) inhibitor, capable of suppressing osteoclast differentiation via downregulation of c-Fos expression (27, 28). To validate this mechanism, we first treated cells with the pan-HDAC inhibitor trichostatin A (TSA), which significantly suppressed osteoclast differentiation (**Supplementary Figures S5A, B**). Subsequent treatment with increasing concentrations of butyric acid resulted in a dose-dependent reduction in NFATC1 protein expression (**Figure 4G**), along with a marked decrease in its mRNA expression (**Figure 4H**). Time-course analysis revealed that butyric acid consistently reduced NFATC1 protein expression throughout osteoclast differentiation (**Figure 4I**). We also observed dose-dependent downregulation of c-Fos protein levels in butyric acid-treated osteoclasts (**Figure 4I**).

Beyond its role HDAC inhibitory effects, butyric acid also modulates host physiology by activating G protein-coupled receptor 43 (GPR43) (29, 30). To assess the involvement of GPR43 in butyric acid-mediated osteoclast inhibition, RAW264.7 cells were treated with the GPR43 antagonist GLPG0974. Interestingly, GLPG0974 alone promoted osteoclast differentiation; however, it failed to reverse the suppression of osteoclast formation by butyric acid treatment at any concentration (**Supplementary Figures S5C, D**).

Collectively, these findings suggest that butyric acid inhibits osteoclastogenesis by suppressing c-Fos expression, thereby attenuating NFATC1 transcription and protein expression. Given that butyric acid levels were significantly increased by 4aa treatment, these data imply that the therapeutic effect of 4aa is closely associated with SCFA-mediated signaling. Moreover, the modulation of microbial taxa such as *Akkermansia* and *Lactobacillus* may contribute to the attenuation of OVX-induced bone loss by promoting the production of bioactive metabolites like butyric acid.

### 4aa modulates gut metabolites and suppresses osteoclast differentiation via α-KIV

**Figure 5:**
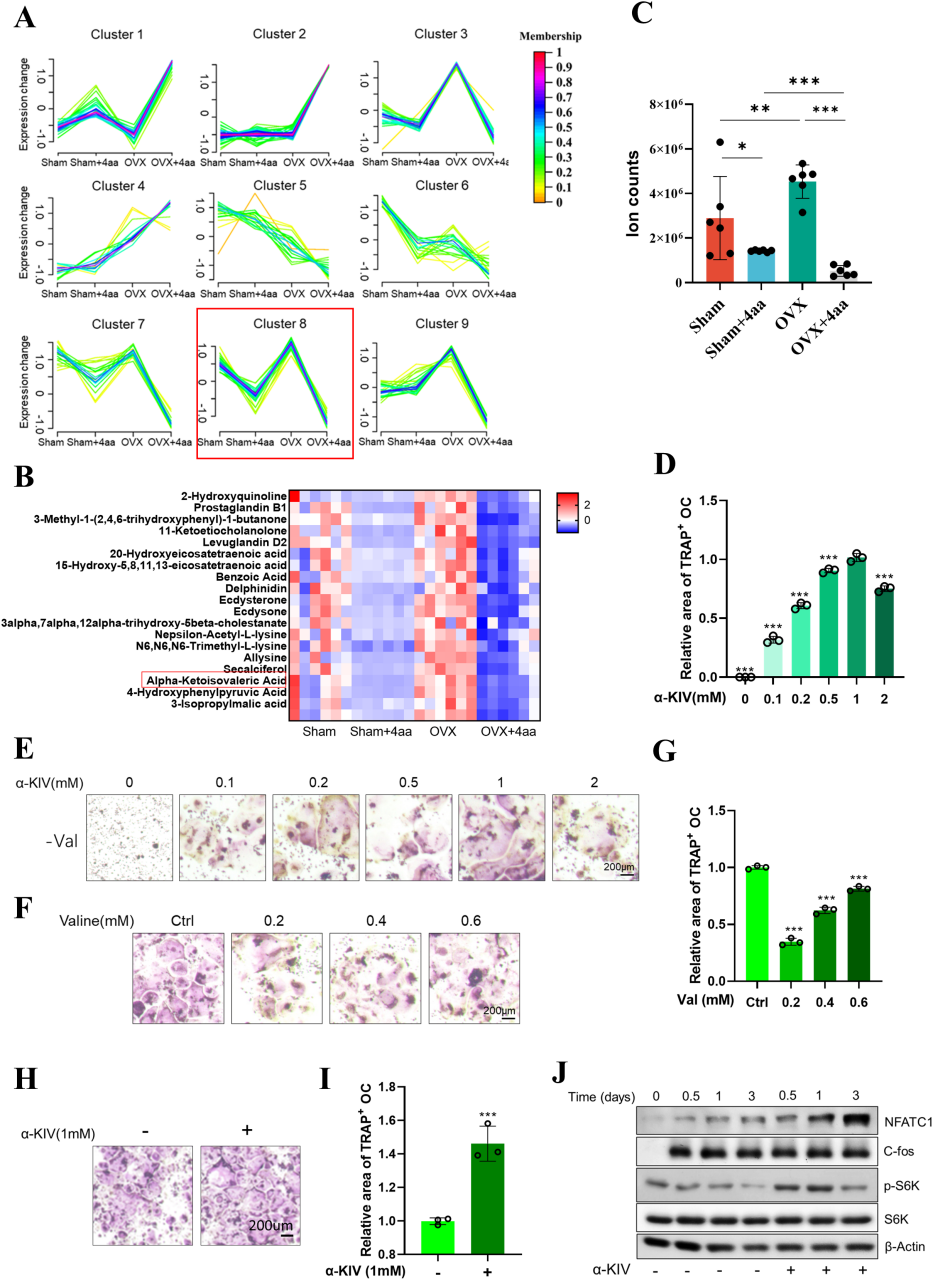
Correlation between the gut microbiota and metabolome in OVX and OVX+4aa groups. **(A)** Metabolite clustering based on taxonomic profiling; **(B)** Heatmap of Cluster 8 metabolites; **(C)** α-KIV levels in gut samples; **(D)** Quantification of TRAP staining in α-KIV–treated RAW264.7 cells under valine deprivation; **(E)** Representative TRAP staining images under valine deprivation with α-KIV; **(F)** TRAP staining with varying concentrations of valine;**(G)** TRAP staining with varying concentrations of valine; **(H, I)** TRAP staining with 1 mM α-KIV; **(J)** Protein expression in differentiating osteoclasts after 1 mM α-KIV. *p < 0.05, **p < 0.01, and ***p < 0.001.

To further investigate how 4aa exerts its anti-osteoporotic effects through modulation of the gut microbiota and its associated metabolites, we performed a categorical analysis of gut metabolites based on their abundance across the different treatment groups (**Figure 5A**). Among the identified clusters, Cluster 8 (highlighted in red) was characterized by decreased metabolite levels following 4aa treatment and increased levels in the OVX group compared to controls (**Figure 5B**), suggesting that the metabolites within this cluster may serve as potential targets of 4aa in preventing osteoporosis.

Within this cluster, the valine-derived metabolite α-KIV was significantly elevated in the OVX group and markedly reduced following 4aa treatment (**Figure 5C**). Valine, a branched-chain amino acid (BCAA), has been previously reported to influence osteoclast differentiation, with valine shown to suppress osteoclastogenesis (31). Consistent with these findings, our study confirmed that valine deprivation significantly inhibited osteoclast differentiation (**Figures 5F, G**); however, the underlying mechanism remained unclear.

To determine whether α-KIV mediates the effects of valine on osteoclast differentiation, α-KIV was supplemented under valine-deprived conditions. Supplementation with α-KIV restored osteoclast differentiation that had been suppressed by valine deprivation (**Figures 5D, E**). Furthermore, α-KIV alone significantly enhanced osteoclast differentiation (**Figures 5H, I**), indicating that this valine-derived metabolite may play a key role in modulating osteoclast activity. Mechanistically, α-KIV treatment led to upregulation of NFATc1, whereas expression of c-Fos, an upstream regulator, remained unchanged (**Figure 5J**). In addition, α-KIV supplementation increased phosphorylation of mTORC1, supporting its role as a metabolic activator of this signaling axis (**Figure 5J**). These findings suggest that α-KIV promotes osteoclast differentiation primarily through upregulation of NFATc1 expression.

### α-KIV-induced osteoclastogenesis depends on BCAT2 and BCKDC

**Figure 6:**
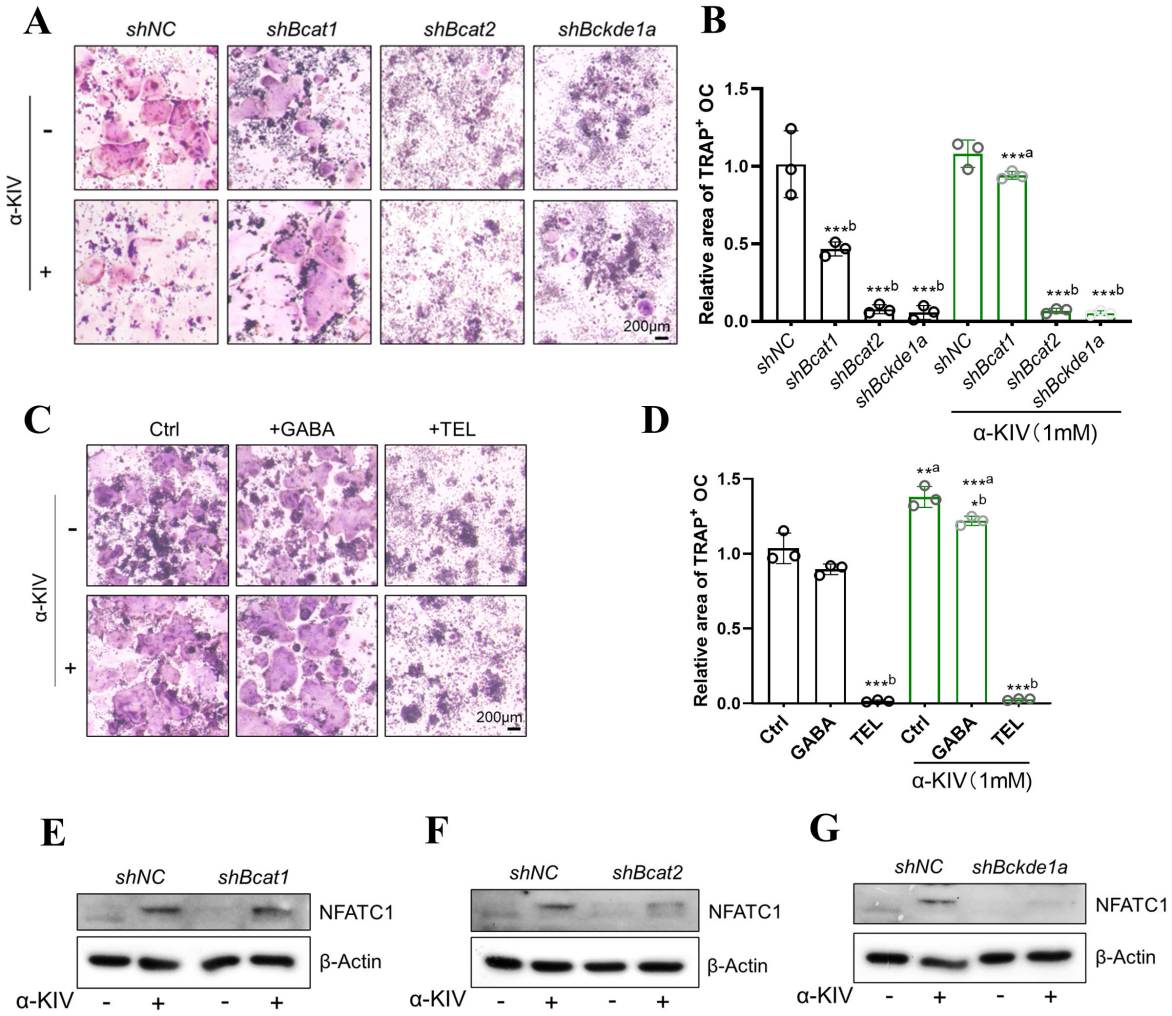
α-KIV–induced osteoclastogenesis requires BCAT2 and BCKDE1a. **(A, B)** TRAP staining and quantification in *shBcat1*, *shBcat2*, and *shBckde1a* cell models under 1 mM α-KIV; **(C, D)** TRAP staining and quantification in α-KIV–treated cells with BCAT1 (gabapentin GABA) and BCAT2 (telmisartan TEL) inhibitors; **(E-G)** NFATc1 protein expression after 3-day α-KIV treatment in knockdown models. Superscript “a” denotes comparison vs untreated control; “b” denotes comparison within treatment condition. *p < 0.05, ***p < 0.01, and ***p < 0.001.

Given the potential involvement of branched-chain amino acid (BCAA) metabolism in α-KIV-mediated signaling, we established knockdown models of BCAT1, BCAT2, and BCKDE1a in RAW264.7 cells. BCAT1 (cytosolic) and BCAT2 (mitochondrial) encode branched-chain aminotransferases that catalyze the reversible transamination of BCAAs to their corresponding keto acids, including α-KIV. BCKDE1a is a catalytic component of the branched-chain α-keto acid dehydrogenase complex (BCKDC), which irreversibly decarboxylates α-KIV, thereby regulating its catabolism. (**Supplementary Figures S5E-H**). Knockdown of Bcat1 significantly impaired osteoclast differentiation; however, α-KIV supplementation successfully rescued this effect (**Figures 6A, B**). In contrast, knockdown of Bcat2 or Bckde1a completely abolished multinucleated osteoclast formation, and α-KIV treatment failed to restore the differentiation capacity in these models (**Figures 6A, B**).

To validate these findings pharmacologically, gabapentin (a BCAT1 inhibitor) and telmisartan (a BCAT2 inhibitor) were used to inhibit enzyme activity during osteoclastogenesis. Gabapentin had no effect on α-KIV–induced osteoclast differentiation, whereas telmisartan significantly inhibited osteoclast formation despite α-KIV supplementation (**Figures 6C, D**).

We further evaluated NFATc1 expression in α-KIV-treated knockdown cells. While shBcat1 cells exhibited restored NFATc1 expression upon α-KIV treatment, no rescue was observed in shBcat2 or shBckde1a cells (**Figures 6E-G**). These results demonstrate that knockdown of BCAT2 or BCKDE1a impairs α-KIV-induced osteoclastogenesis and suggest that the promotive effect of α-KIV on osteoclast differentiation is dependent on BCAT2 and BCKDC.

### Effect of gut microbiota on 4aa alleviation of osteoporosis in mice

**Figure 7:**
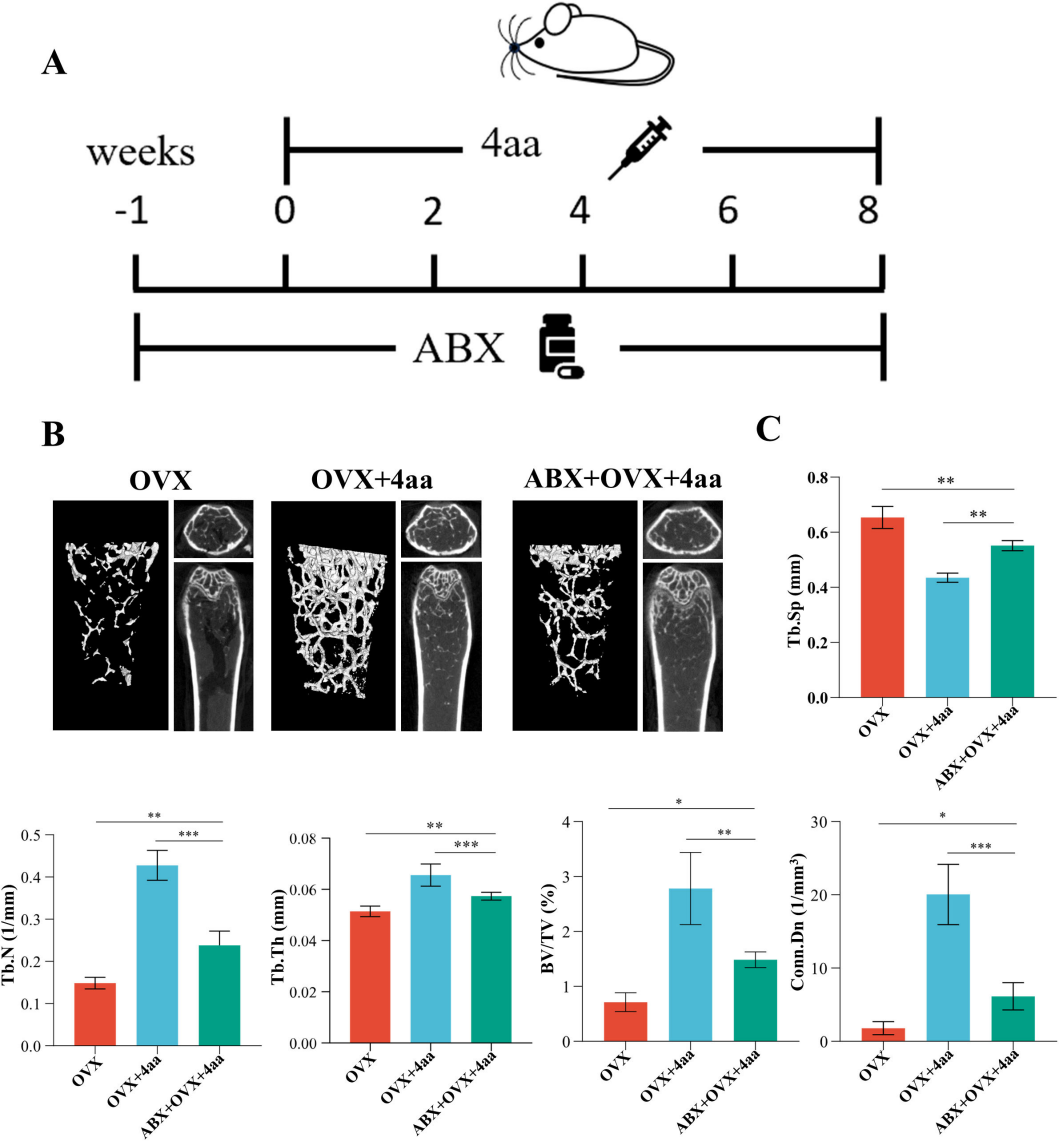
Role of gut microbiota in 4aa-mediated protection against osteoporosis. **(A)** Antibiotic (ABX) treatment schematic; **(B)** Representative 3D micro-CT post-ABX in OVX and 4aa-treated mice; **(C)** Statistical analysis of trabecular bone volume fraction (BV/TV), trabecular junction density (Conn.Dn), trabecular number (Tb.N), trabecular space (Tb.Sp), and trabecular thickness (Tb.Th) across OVX, OVX+4aa, and OVX+4aa+ABX groups (N=6). *p<0.05, **p<0.01, and ***p<0.001.

Our previous results have shown demonstrated that treatment with 4aa altered the gut microbiota composition in mice. To verify the contribution of the gut microbiota to the anti-osteoporotic effects of 4aa, we administered a broad-spectrum antibiotic cocktail (ABX) in drinking water prior to and during intraperitoneal injection of 4aa (**Figure 7A**). A previous study reported that ABX treatment significantly reduced the ACE, Shannon, and Simpson indices of gut microbial diversity (32), indicating decreased species richness and diversity. Principal component analysis (PCA) revealed that the microbiota composition in the ABX-treated groups clustered distinctly from the Sham and OVX groups, confirming effective depletion of the gut microbiota (33).

We observed that the anti-osteoporotic effect of 4aa was attenuated following ABX treatment; however, femoral bone mass remained significantly higher than in the OVX group (**Figure 7B**). Additionally, parameters including Tb.Sp, trabecular joint density, BV/TV, Tb.N, and Tb.Th were significantly decreased (**Figure 7C**). These findings suggest that while the gut microbiota contributes to the full therapeutic efficacy of 4aa, microbiota-independent mechanisms may also be involved. This is consistent with previous studies showing that antibiotic-induced dysbiosis impairs bone homeostasis through microbiota-immune interactions and disrupted metabolic signaling pathways (34, 35).

## Discussion

Osteoporosis is a common condition associated with serious health risks, including fracture rates and related complications. However, current treatment strategies have limited efficacy and are often accompanied by significant adverse effects, highlighting the urgent need for safer and more effective therapies. Building on our previous study demonstrating that 4aa inhibits osteoclastogenesis and promotes osteogenesis (20), we now provide evidence that 4aa alleviates osteoporosis by modulating the composition and abundance of the gut microbiota, suggesting an alternative therapeutic approach.

Previous research indicates that dysbiosis of the gut microbiota impairs bone metabolism, and certain drugs, such as glucocorticoids (GCs), induce bone loss through disruption of microbial communities (36, 37). In our study, 4aa treatment resulted in significant changes in the gut microbiota of OVX mice. Notably, the genus *Akkermansia* has been positively correlated with improvements in bone volume, length, and strength parameters, and supplementation with *Bacillus* spp. has previously been reported to enhance BMD, bone mineral concentration (BMC), and cortical thickness in OVX models (38–40). Consistent with these studies, we observed a marked increase in the relative abundances of *Akkermansia* and *Bacillus* spp. following 4aa administration in OVX mice, whereas these microbial genera remained unchanged in sham group, suggesting that these taxa may contribute to the bone-preserving effects of 4aa in estrogen-deficient conditions.

Gut microbiota-derived metabolites represent pivotal mediators in host-microbe interactions that significantly influence bone homeostasis (41–43). Our metabolomic analyses revealed substantial alterations in fecal metabolites following 4aa treatment in OVX mice, particularly increases in beneficial metabolites such as citronellal and kaempferol. Citronellal has been shown to reduce mitochondrial oxidative stress and endothelial dysfunction (44, 45), whereas kaempferol promotes bone formation by enhancing BMD and suppressing osteoclastogenic markers (46–48). Correspondingly, we found a significant increase in microbial production of kaempferol following 4aa treatment compared to controls. Additionally, levels of the osteoclast-promoting metabolite chlorpromazine (CLP), known to reduce trabecular bone volume and strength (49), decreased significantly with 4aa administration. Correlation analysis further suggested that increased abundances of *Akkermansia* and *Bacillus* spp were were associated with elevated levels of kaempferol, butyric acid, and citronellal, implying a potential role for these bacterial taxa in metabolic reprogramming. Specifically, butyric acid was confirmed to suppress osteoclastogenesis through downregulation of c-Fos and NFATc1 expression. Members of the genus Bacillus may potentially contribute by producing these bioactive metabolites (50–53). In contrast, *Dubosiella* was correlated with detrimental metabolites. Nonetheless, direct causative relationships between these bacterial taxa and the metabolites, and their roles in bone metabolism, require further investigation.

4aa primarily consists of selenium and a trifluoroethoxy group. Many studies have shown that one-quarter of microorganisms express selenoproteins, and some bacteria even require selenium for optimal growth, while others are sensitive to its toxicity depending on their redox environment and genetic background (19, 52). Selenium not only exerts direct selective pressure on gut microbial composition but also plays a key role in regulating intestinal immunity and epithelial barrier function. In the cecum, selenium modulates inflammatory cytokines such as TNF-α, IL-1β, and TGF-β1, and its supplementation has been shown to alleviate mucosal inflammation and promote microbial homeostasis (53, 54). Selenium also enhances the expression of junctional adhesion molecules (JAMs), reinforcing intestinal barrier integrity, which is crucial in maintaining gut–bone axis stability (55–57). In addition to selenium, the trifluoroethoxy group in 4aa is a well-recognized pharmacophore known to enhance drug specificity and bioavailability. For instance, the introduction of a trifluoroethoxy group into selective α1-adrenergic receptor blockers increased receptor selectivity by 38-fold (58, 59). We hypothesize that in 4aa, this moiety may enhance drug accumulation or activity specifically at the gut interface, thereby amplifying selenium’s bioactivity at the target site. Therefore, the combined actions of selenium and trifluoroethoxy modification may enable 4aa to modulate the gut microbiota with enhanced precision-both by directly shifting microbial composition and by suppressing local inflammation and restoring mucosal integrity. These synergistic effects are likely critical in mediating the osteoprotective outcomes observed in OVX mice.

Importantly, our study is the first to demonstrate that 4aa inhibits osteoclast differentiation through suppression of α-KIV accumulation in the gut. *In vitro* experiments demonstrated that α-KIV promotes osteoclast differentiation primarily by enhancing NFATc1 protein expression. α-KIV treatment also increased phosphorylation of p70-S6K, a downstream effector of the mTORC1 pathway. However, previous studies have indicated that hyperactivation of mTORC1 may suppress osteoclastogenesis (60, 61), suggesting complex, possibly dual regulatory roles for mTORC1 in osteoclast differentiation. Thus, it remains unclear whether α-KIV exerts its osteoclast-promoting effect primarily via mTORC1 activation, or through an mTORC1-independent pathway affecting NFATc1 expression. Mechanistically, we also observed that the pro-osteoclastogenic effects of α-KIV were significantly reduced in BCAT2- or BCKDC-knockdown models. Considering that both enzymes also regulate the metabolism of other branched-chain amino acids (BCAAs), such as leucine and isoleucine, further research is necessary to determine whether impaired osteoclast differentiation results specifically from disrupted α-KIV metabolism or more broadly from disturbances in overall BCAA metabolic pathways.

Despite the promising therapeutic potential of 4aa, several critical aspects remain unresolved and require further investigation. Foremost, comprehensive pharmacokinetic and safety profiles of 4aa have yet to be characterized. Essential parameters such as maximal concentration (C_max), half-life, and metabolic fate *in vivo* are currently unknown, as are potential long-term systemic toxicities-including hepatic and renal dysfunction and selenium accumulation. Addressing these safety considerations will be critical for establishing a therapeutic window and ensuring clinical safety of the compound. Additionally, although beneficial microbial taxa such as *Akkermansia muciniphila* were enriched following 4aa treatment, their causal contribution to skeletal benefits requires confirmation through experimental approaches such as fecal microbiota transplantation (FMT) and mono-colonization studies. Furthermore, the specific microbial species responsible for generating key metabolites like butyrate or α-KIV remain to be isolated and functionally validated. Employing culture-dependent and functional metagenomic methods will be necessary to clarify microbial sources and metabolic pathways involved. Lastly, potential synergistic effects of combining 4aa with dietary prebiotics (e.g., inulin) or established anti-osteoporotic medications have not yet been explored. Investigating these combinations may provide novel therapeutic insights and enhanced clinical effectiveness in managing osteoporosis.

Addressing these knowledge gaps through targeted research will significantly enhance our understanding of the mechanisms underlying the beneficial effects of 4aa and accelerate its development as a potential therapeutic candidate for osteoporosis.

## Conclusion

This study demonstrated that 4aa, a novel compound, prevents osteoporosis by modulating gut microbiota composition and associated metabolites. These findings provide a preclinical foundation for the potential therapeutic application of 4aa in osteoporosis.

## Data Availability

The 16S rRNA sequencing data supporting the findings of this study have been submitted to the NCBI Sequence Read Archive (SRA) under the accession number PRJNA1284016. The associated intestinal metabolomic data are provided in **Supplementary Material 2**. All data supporting the findings of this study are available from the corresponding authors upon reasonable request.

## Supplementary material

Supplementary Figure 1
**(A)** 3D micro-CT images of OVX mice treated with various 4aa concentrations; **(B)** Quantification of trabecular bone volume fraction (BV/TV), trabecular junction density (Conn.Dn), trabecular number (Tb.N), trabecular space (Tb.Sp), and trabecular thickness (Tb.Th) between the OVX and OVX+4aa groups, N=6. *p<0.05, **p<0.01, and ***p<0.001.

Supplementary Figure 2
**(A–J)** Relative abundance of bacterial phyla across groups (N=6). *p<0.05, **p<0.01, and ***p<0.001.

Supplementary Figure 3
**(A)** Top 10 elevated fecal metabolites in OVX+4aa mice; **(B)** Top 10 reduced fecal metabolites. *p<0.05, **p<0.01, and ***p<0.001.

Supplementary Figure 4
**(A)** Correlation matrix of microbial genera and fecal metabolites.

Supplementary Figure 5
**(A, B)** TRAP staining of RAW264.7 cells treated with butyric acid (0.5 mM) and HDAC inhibitor (Trichostatin A: TSA, 5 nM) for 4 days; **(C, D)** TRAP staining and quantitative analysis following treatment with the GPR43 inhibitor (GLPG0974), with or without butyric acid; **(E)** Quantitative real-time PCR verifying knockdown efficiency; **(F-H)** Western blot analysis showing protein-level knockdown efficiency of BCAT1, BCAT2, and BCKDE1a. Superscript “a” denotes comparison vs untreated control; “b” denotes comparison within treatment condition. *p < 0.05, ***p < 0.01, and ***p < 0.001.
