# Supplementary figures and images for: The novel organoselenium compound 4aa ameliorates osteoporosis by modulating gut microbiota composition and fecal metabolite profiles
Source: Front Endocrinol (Lausanne). 2025 Aug 13;16:1623933. doi: 10.3389/fendo.2025.1623933 (PMC12380565; doi:10.3389/fendo.2025.1623933)

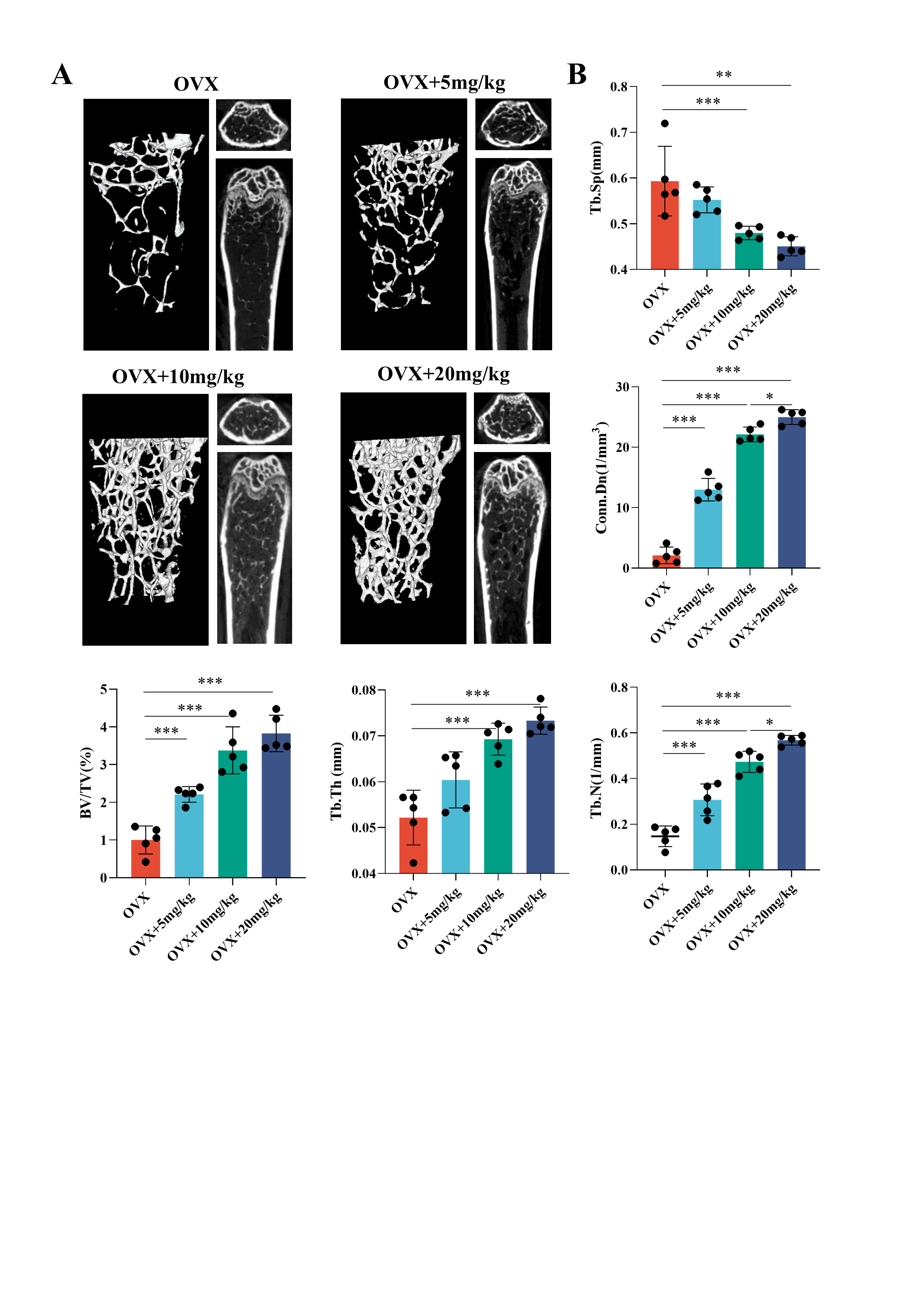

Supplement: Supplementary Figure 1 — (A) 3D micro-CT images of OVX mice treated with various 4aa concentrations; (B) Quantification of trabecular bone volume fraction (BV/TV), trabecular junction density (Conn.Dn), trabecular number (Tb.N), trabecular space (Tb.Sp), and trabecular thickness (Tb.Th) between the OVX and OVX+4aa groups, N=6. *p<0.05, **p<0.01, and ***p<0.001. [file Image1.tif]

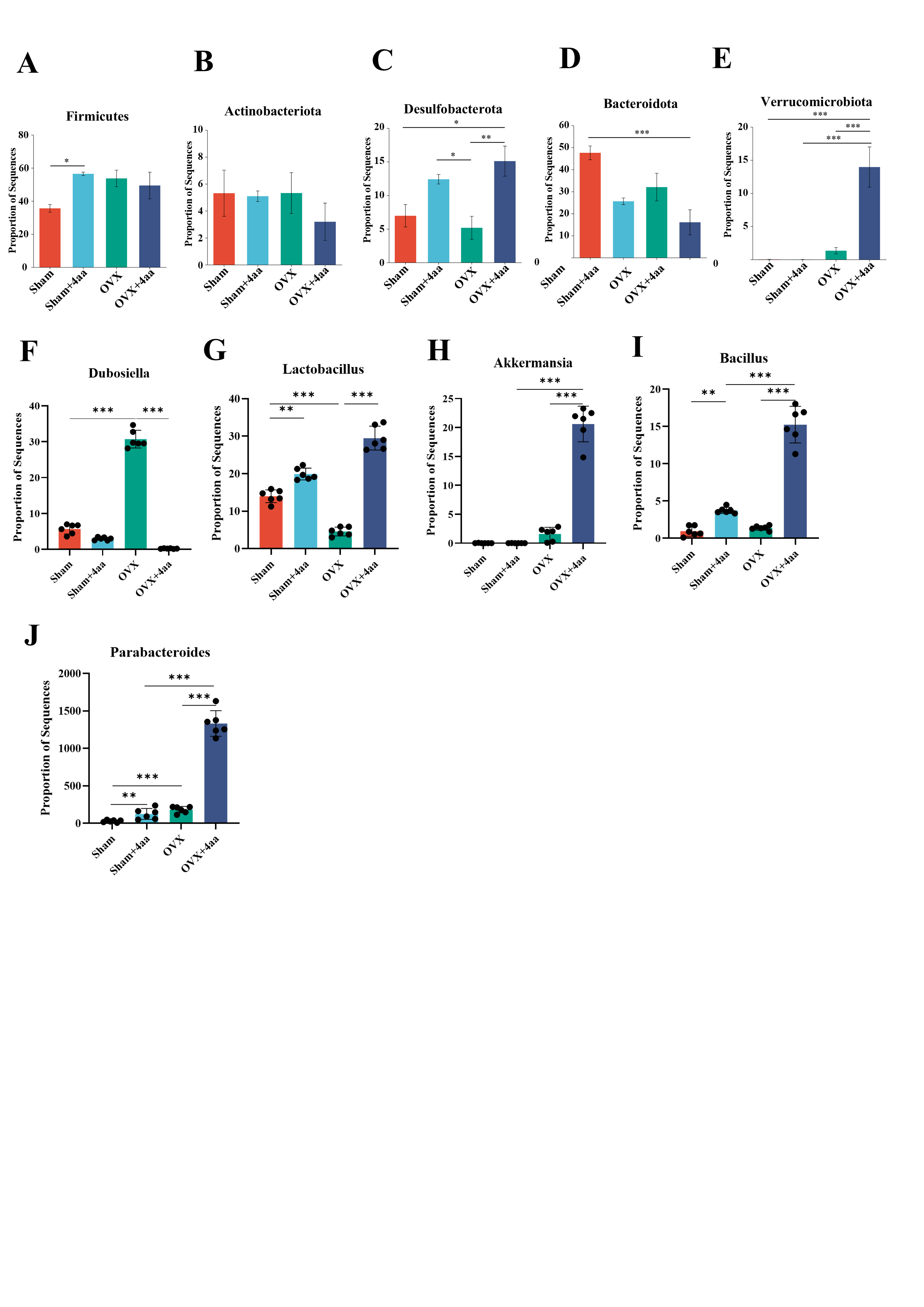

Supplement: Supplementary Figure 2 — (A–J) Relative abundance of bacterial phyla across groups (N=6). *p<0.05, **p<0.01, and ***p<0.001. [file Image2.tif]

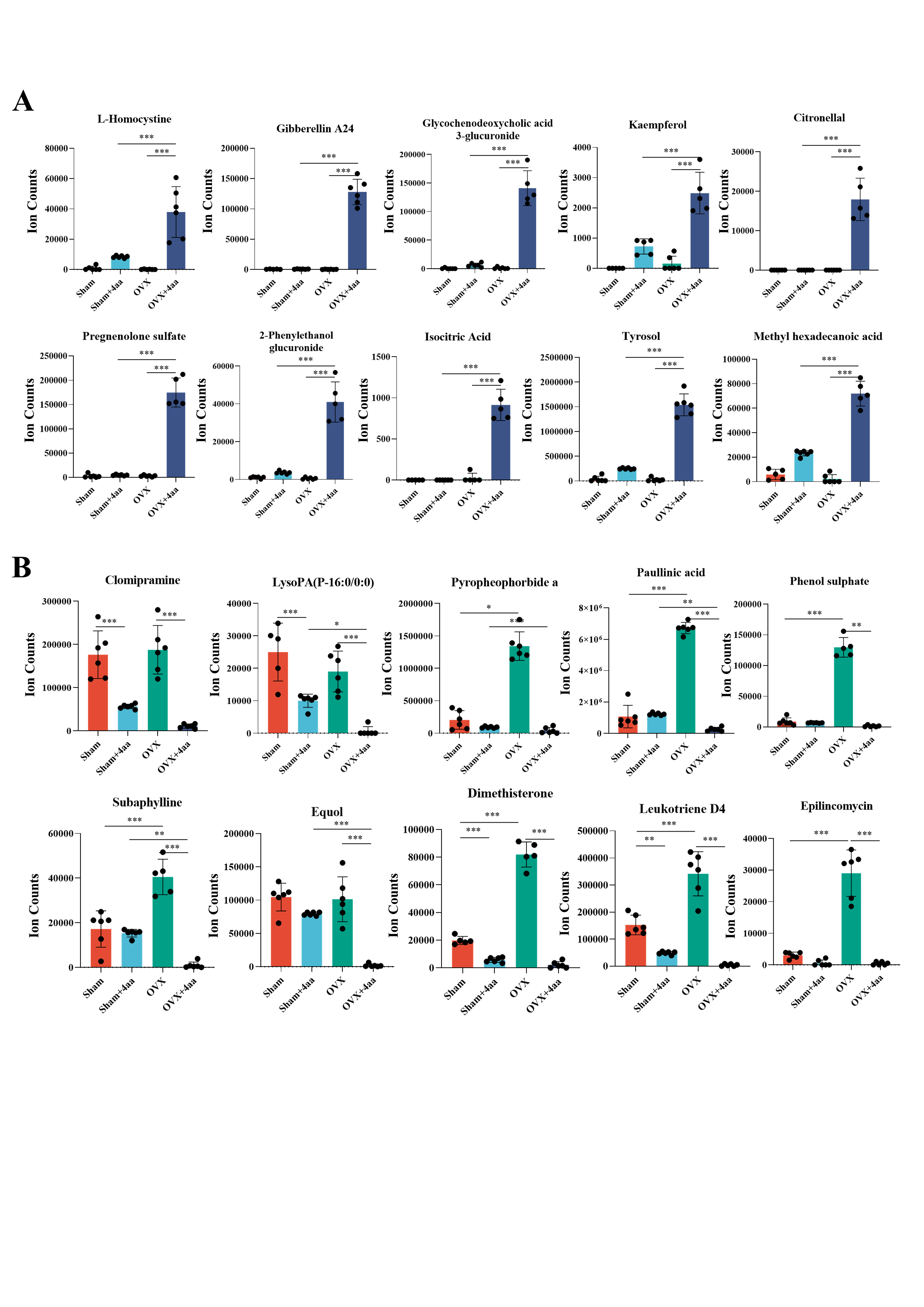

Supplement: Supplementary Figure 3 — (A) Top 10 elevated fecal metabolites in OVX+4aa mice; (B) Top 10 reduced fecal metabolites. *p<0.05, **p<0.01, and ***p<0.001. [file Image3.tif]

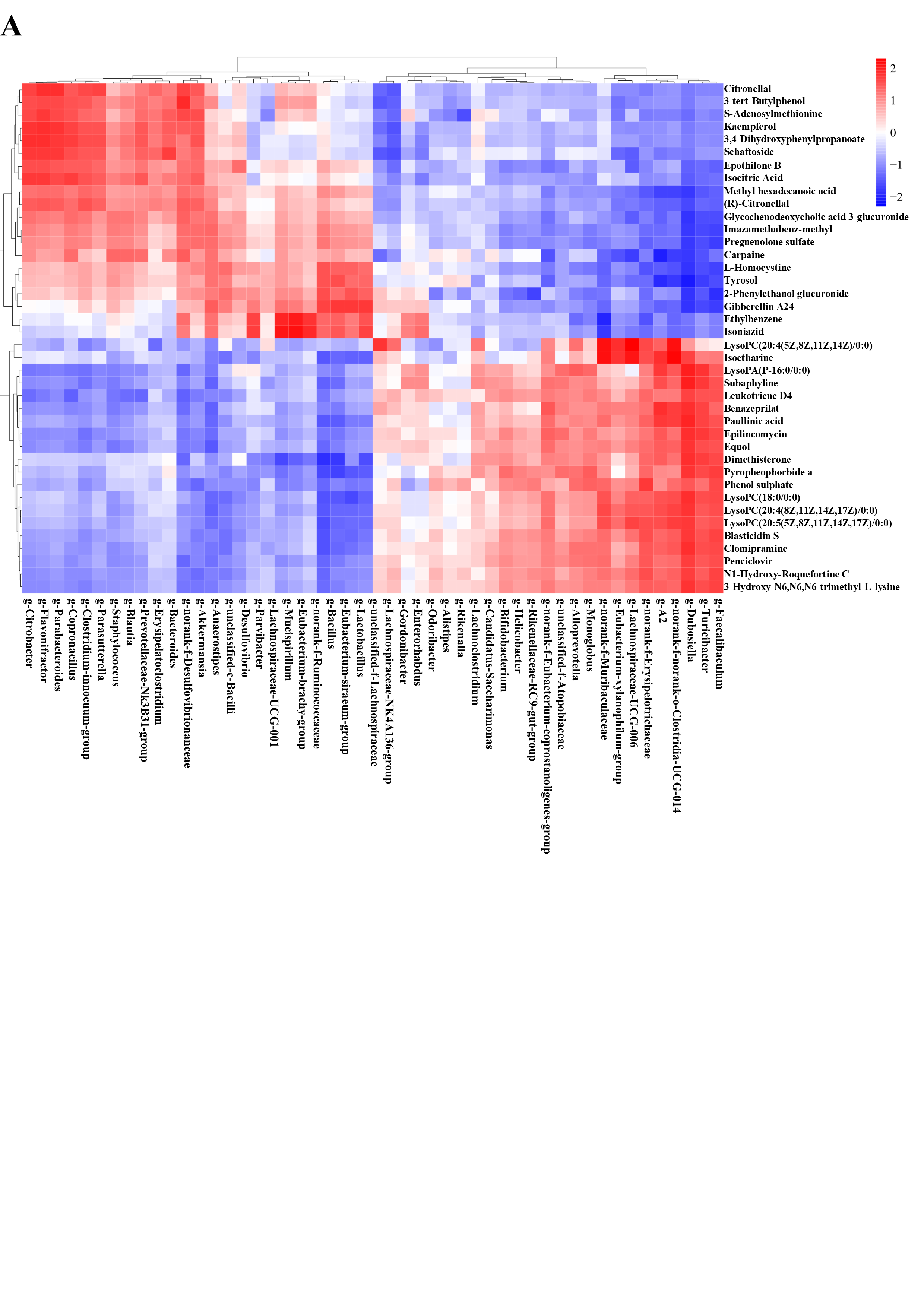

Supplement: Supplementary Figure 4 — (A) Correlation matrix of microbial genera and fecal metabolites. [file Image4.tif]

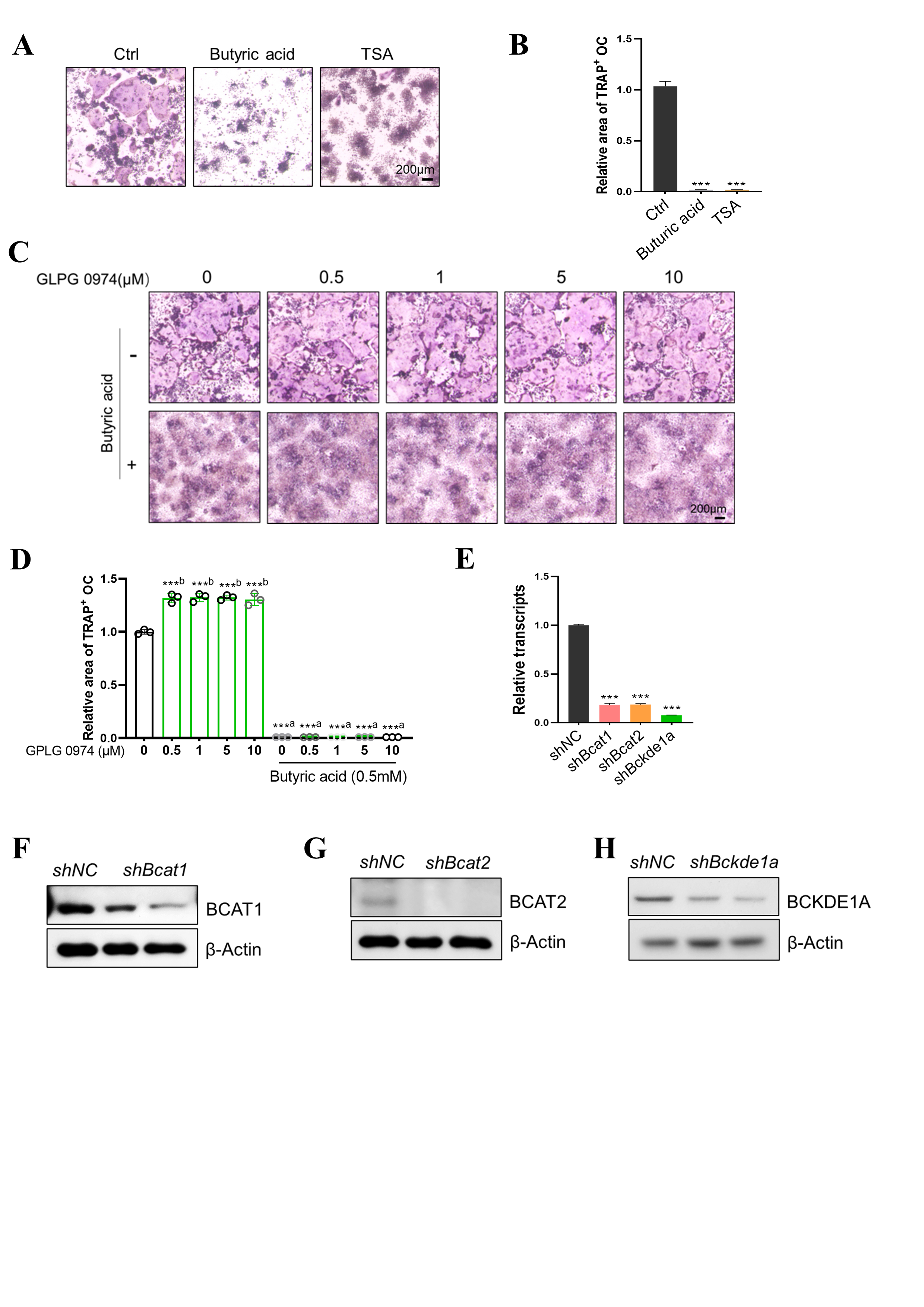

Supplement: Supplementary Figure 5 — (A, B) TRAP staining of RAW264.7 cells treated with butyric acid (0.5 mM) and HDAC inhibitor (Trichostatin A: TSA, 5 nM) for 4 days; (C, D) TRAP staining and quantitative analysis following treatment with the GPR43 inhibitor (GLPG0974), with or without butyric acid; (E) Quantitative real-time PCR verifying knockdown efficiency; (F-H) Western blot analysis showing protein-level knockdown efficiency of BCAT1, BCAT2, and BCKDE1a. Superscript “a” denotes comparison vs untreated control; “b” denotes comparison within treatment condition. *p < 0.05, ***p < 0.01, and ***p < 0.001. [file Image5.tif]

Fig 4G

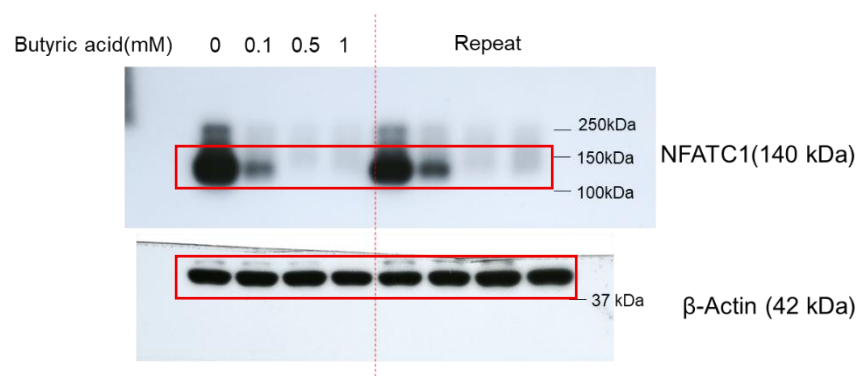

Fig 4I

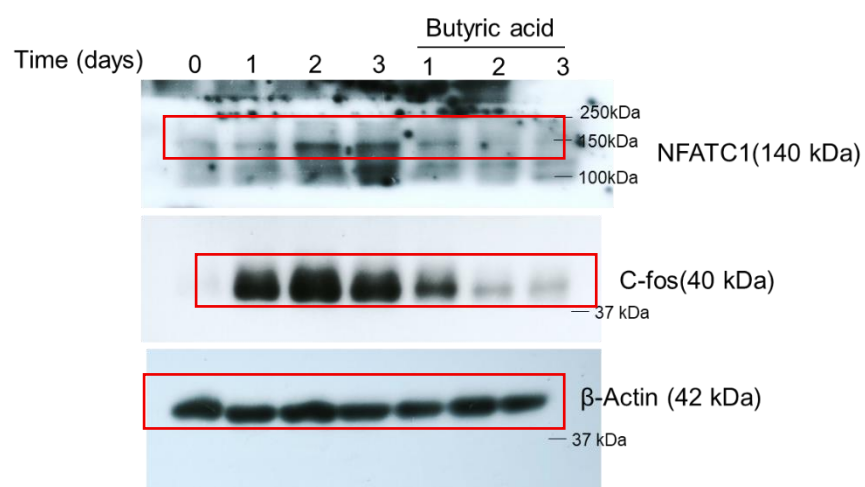

Repeat

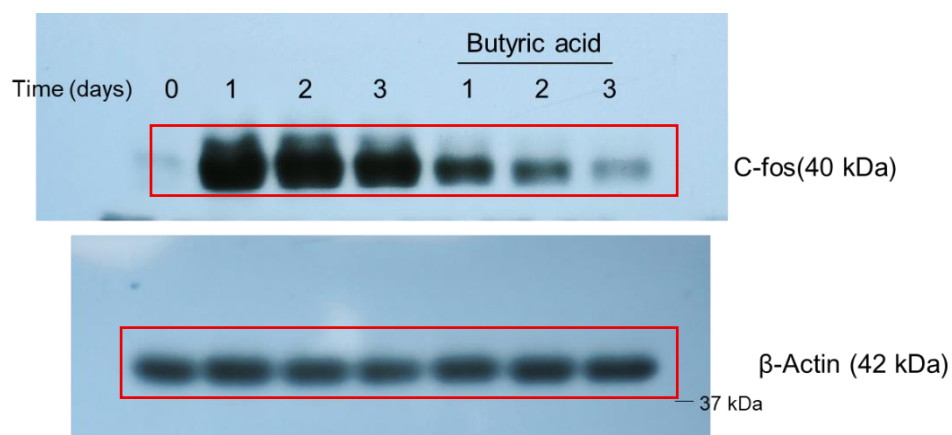

Fig 5J

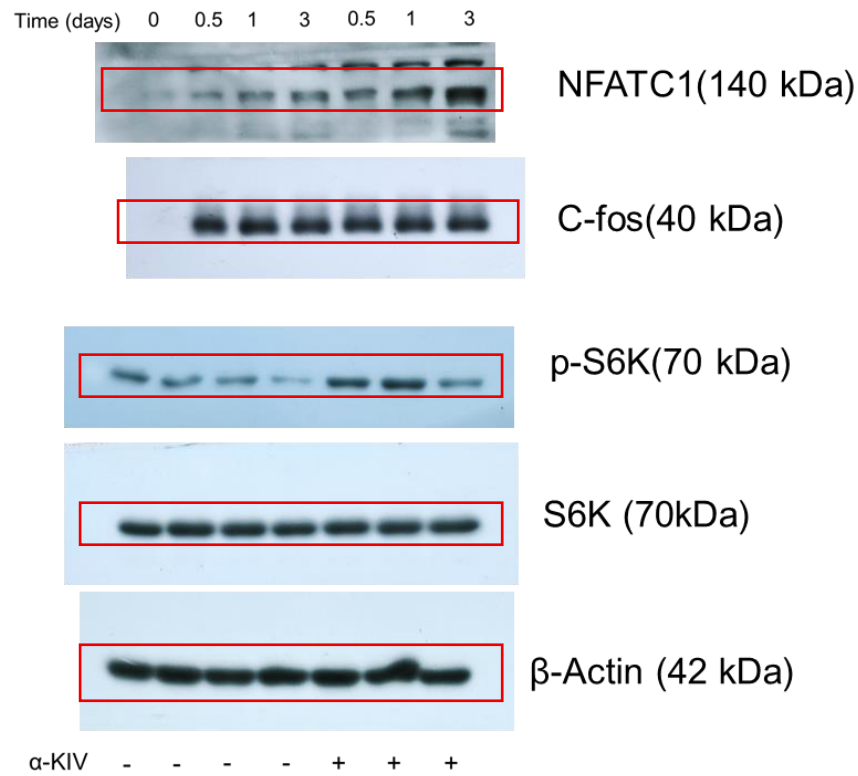

**Repeat**

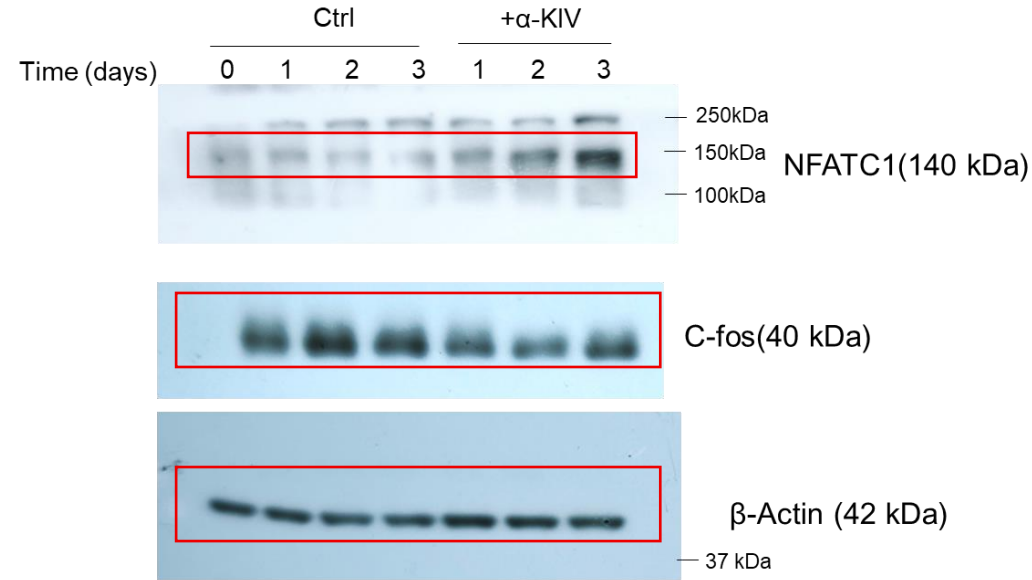

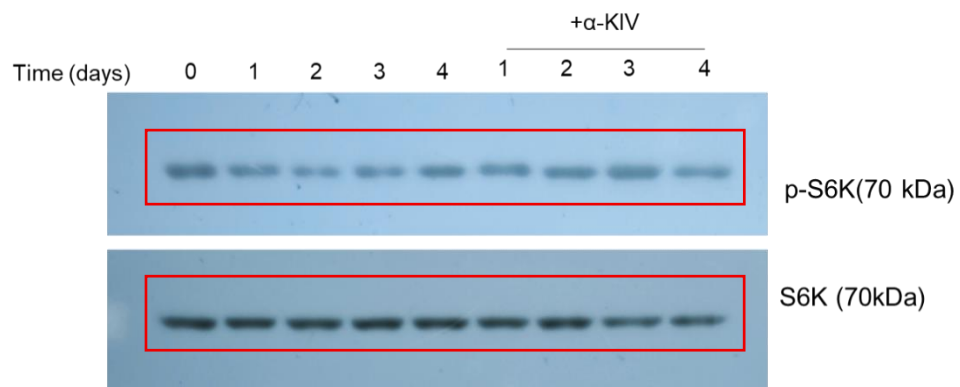

Fig6 E-G

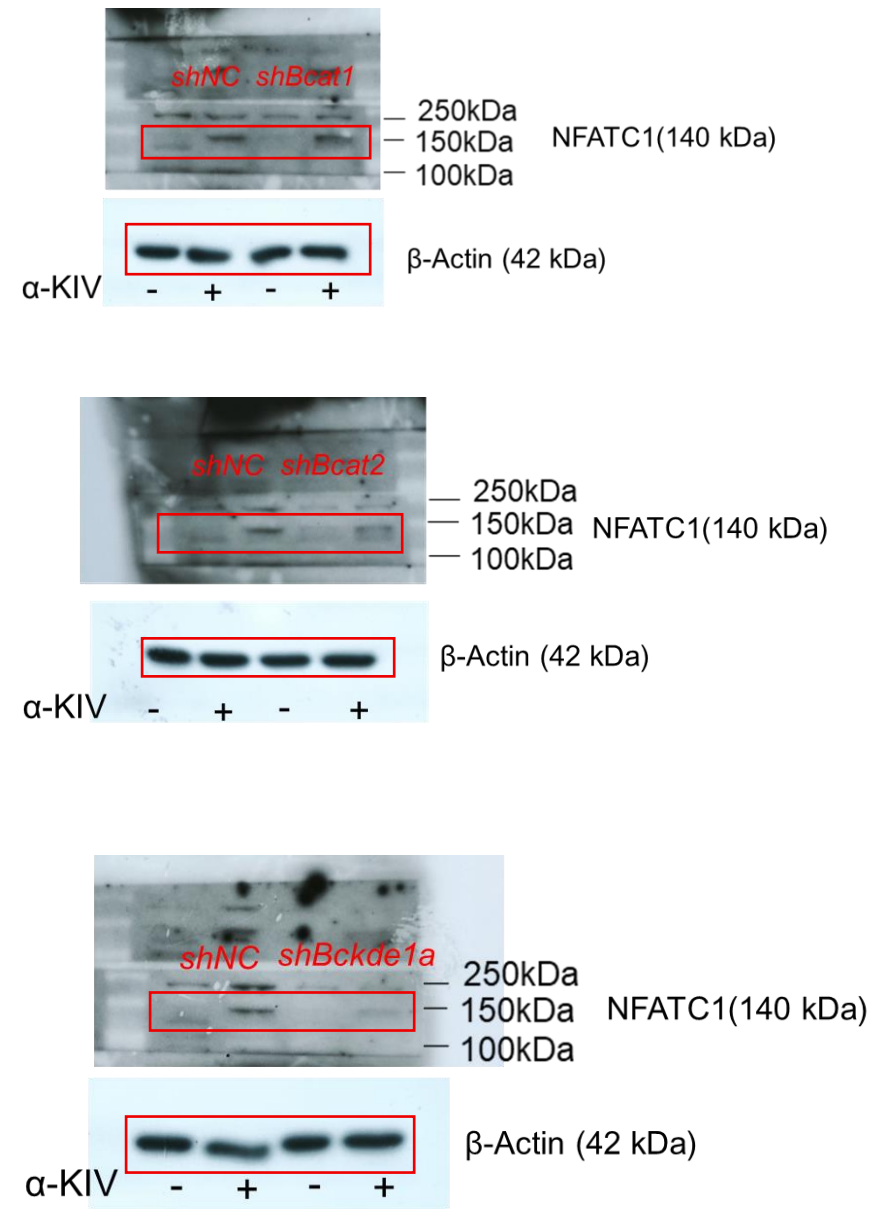

Repeat

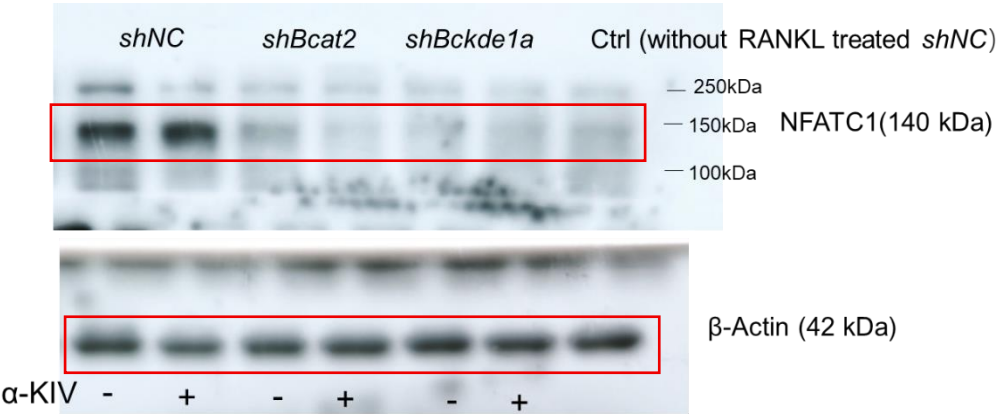

Fig S6 E-H

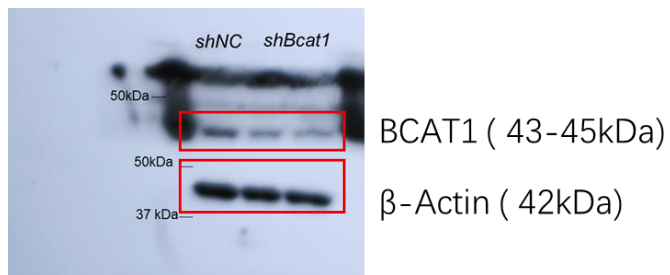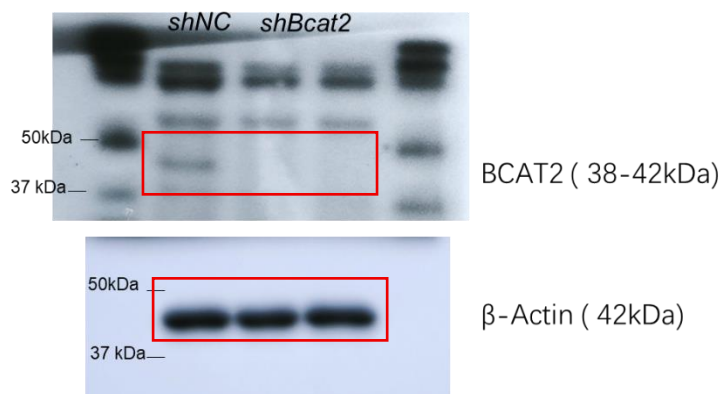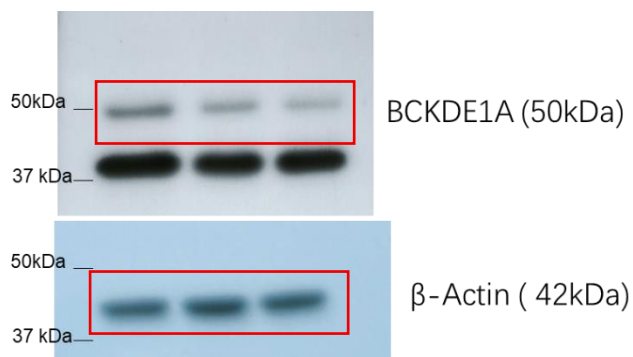

Supplement: Supplementary file 8 [file DataSheet3.pdf]
