# Supplementary material for: The novel organoselenium compound 4aa ameliorates osteoporosis by modulating gut microbiota composition and fecal metabolite profiles
Source: Front Endocrinol (Lausanne). 2025 Aug 13;16:1623933. doi: 10.3389/fendo.2025.1623933 (PMC12380565; doi:10.3389/fendo.2025.1623933)
Supplement: Supplementary file 6 [file DataSheet1.pdf]

Supplementary Table 1

Gene	Forward Primer (5'→3')	Reverse Primer (5'→3')
Bcat1	GAAGTGGCGGAGACTTTTAGG	TGGTCAGTAAACGTAGCTCCA
Bcat2	CAAAGGTGGAGACCAGCAGGTA	TGGCGGATACACTCCAACAGCT
Bckdha	CTCCTGTTGGGACGATCTGG	CATTGGGCTGGATGAACTCAA
β-Actin	CATCCGTAAAGACCTCTATGCCAAC	ATGGAGCCACCGATCCACA

Supplementary Table 2

Antibody	Manufacturer	Catalog No.	Dilution
β-Actin	Proteintech	66009-1-Ig	1:1000
BCAT1	Proteintech	13640-1-AP	1:1000
BCAT2	Proteintech	16417-1-AP	1:1000
BCKDE1a	Santa Cruz Biotechnology	sc-271538	1:1000
C-Fos	Santa Cruz Biotechnology	sc-271243	1:1000
NFATC1	Cell signaling technology	8032	1:1000
p70 S6K	Cell signaling technology	34475	1:1000
p70 S6K (Thr389)	Cell signaling technology	9234	1:1000

Supplementary Table 3

Regent	Manufacturer	Catalog No.
Telmisartan	Aladdin Scientific	T129239-1g
Gabapentin	Aladdin Scientific	G122413
GLPG 0974	MedChem Express	HY-12940
Trichostatin A	MedChem Express	HY-15144
Puromycin	Beyotime Biotechnology	ST551
Butyric acid	Aladdin Scientific	B110439
α-KIV	Aladdin Scientific	1460-34-0
